# Effect of heat treatment atmospheres on microstructure evolution and corrosion resistance of 2205 duplex stainless steel weldments

**DOI:** 10.1038/s41598-023-31803-5

**Published:** 2023-03-21

**Authors:** Abdelkarim Yousif Mohamed, Ahmed Hussein Abdelraheem Mohamed, Z. Abdel Hamid, Ahmed Ismail Zaky Farahat, A. E. El-Nikhaily

**Affiliations:** 1 Mechanical Production Department, Faculty of Technology & Education, Suez University, Suez, 43527 Egypt; 2 Central Metallurgical Research and Development Institute (CMRDI), Cairo, Egypt

**Keywords:** Chemistry, Engineering, Materials science

## Abstract

The effects of post heat treatment atmosphere on microstructure and corrosion resistance of duplex stainless steel welded joints were investigated. Post weld heat treatment (PWHT) was carried out with and without protective atmospheres. Nitrogen and argon are used as protective gases individually. Detailed microstructure examination (optical and SEM) demonstrates that nitrides precipitates are highly observed in the welded zones for nitrogen protected samples. An observed drop of ferrite volume fraction in post weld heat treated samples compared with welded samples without heat treatment leading to corrosion resistance enhancement of heat treated welded joints. An exception for using nitrogen as heat treatment atmosphere a decreased corrosion resistance of weldments is investigated due to nitride precipitates. An increase in the weld zone hardness for post weld heat treated samples compared with base alloy. The initial hardness of duplex stainless steel was 286 Hv while average hardness of weld zone was 340, 411, 343, and 391 Hv for as welded, PWHT using air, argon, and nitrogen atmospheres, respectively. Weld zone hardness increased to 33, 44, 20, and 37%. A significant decrease in the ultimate tensile strength and elongation after PWHT. The initial Ultimate tensile strength duplex stainless steel base material was 734.9 MPa while Ultimate tensile strength of the welded joints was 769.3, 628.4, 737.8, and 681.4 MPa for the following conditions: as welded, PWHT using air, argon, and nitrogen atmospheres, respectively.

## Introduction

Duplex stainless steel (DSS) is the most suitable metal to use in severe environments such as deep-sea pipelines to transfer petroleum material, seawater desalination, reactors, petroleum tanker, oil refinery chemical, and petrochemical industries owing to its outstanding corrosion resistance and high strength^1,2^. The chemical composition for duplex stainless steel (DSS) contains Cr, Mo, Ni, and N, in addition, the alloying elements distribution of duplex stainless steel (DSS) are inhomogeneous, whereas Cr and Mo are leads to an increase in the ferrite volume fraction, Ni and N increase the austenite volume fraction. The key factor to influence ferrite volume fraction and intermetallic phases precipitation (harmful phases)such as the sigma (σ) phase, chi (χ) phase, secondary austenite (γ2), nitride (CrN and Cr2N), carbides (M_23_C_6_) are annealing temperature, cooling rate, solidification after the welding process^3–8^ and heat input^8–10^.

The welding process is a basic and indispensable process in the industry. It’s a heat treatment process that results in three zones: base material (BM), heat affected zone (HAZ), and weld zone (WZ) each^11^. DSS after welding shows three different zones in chemical composition of ferrite and austenite phase which consequently lead to different corrosion resistance. The welding influence not just be restricted to chemical composition, affect also the volume fraction of ferrite because of heat^12,13^. Whereas Nilsson^14^ indicated the multi pass welding allows to form excessive amount of secondary austenite Thus, it leads to low corrosion resistance in the weld zone.

Furthermore, the selection of the welding electrode is of paramount importance in controlling the microstructure of the welding area and thus on the properties after welding^15–17^. An attempt has been made to investigate the effect of filler metal on solidification, microstructure, and mechanical properties of the dissimilar weld between super duplex stainless 2507 and high strength low alloy API X70 pipeline steel by Khan et al.^15^. They concluded that the 309L filler weld’s microstructure is composed of skeletal ferrites in the austenite matrix, whereas the 2594 filler weld has multiple reformed austenite embedded in the ferrite matrix. Moreover, Ramkumar et al.^18^ investigate the weldability, metallurgical and mechanical properties of the UNS 32750 super- duplex stainless steels joints by Gas Tungsten Arc Welding (GTAW) employing ER2553 and ERNiCrMo-4 filler metals. They recommended the using of ER 2553 for welding super-duplex stainless steel because the enhancement of mechanical properties of welded joints employing ER 2553 compared with welded joints employing ER NiCrMo-4. They attributed this comparative improvement of mechanical properties to the presence of sufficient amounts of ferrite, allotriomorphic and the austenite in the form of wedge shaped widmanstätten and as Intergranular precipitates in the weld zone employing ER2553.

Based on these studies the need for controlling the weld zone microstructure is an important concern. After welding processes, the most famous and important way for microstructure improvement is the post weld heat treatment (PWHT). For proper (PWHT) the following variables should be controlled: heating temperature, holding time, cooling rate, and process atmosphere (protection gas). Post weld heat treatment (PWHT), improper annealing temperature, protective gas, and slow cooling rate promotes formation of intermetallic precipitation (harmful phases) which depends on presence of Cr, Mo, and C.

Several studied were carried out concerning the PWHT of welds in general and duplex stainless steel weldments in particular.

Several studies indicate that the appropriate temperature for annealing is between 1000 and 1200 ℃ followed by water quenching^8,19–21^. On the other hand, Shen^19^ indicated that the optimal annealing temperature for DSS without the presence of intermetallic precipitation is between 1050 and 1100 °C. Whereas Zhang^8^ clarified the effects of short time heat treatment after welding at (a small scale from the temperature where choice) 1020, 1050, 1080, 1100 and 1150 °C and confirmed the optimal annealing temperatures (no intermetallic) are 1050 °C and 1080 °C. In addition, the highest pitting corrosion resistance was at annealing temperature 1080 °C for 3 min.

During PWHT the time of annealing and cooling rate should be considered. Several studies indicate the effects of Solution Annealing Time (holding time) and confirmed that increasing the annealing time leads to a decrease in corrosion resistance^22,23^. While that the cooling rate during PWHT is an important concern that the slower cooling rate after solution treatment of welded joints leads to the formation of harmful phases^24,25^.

Furthermore, several studies showed sigma phase enriched with Cr, Mo, and presence of intermetallic phases (harmful phases) make duplex stainless steel was prone to Embrittlement^4^, and consequently low corrosion resistance and deteriorated mechanical properties^19,20,23,26,27^.

Therefore, the PWHT procedure must be observed carefully to avoid the formation of harmful phases. On the other hand, the proper PWHT can enhance the corrosion resistance of the duplex stainless steel weldments due to the increasing of austenite volume fraction^21^. From this point of view, there is great importance in choosing the optimal conditions for PWHT.

Because few studies were carried out on the effect of heat treatment atmosphere on the microstructure of DSS welded joints, therefore, it was excited to study in details effect of different protective atmospheres (argon and nitrogen) during PWHT on microstructure, mechanical properties, and corrosion resistance of DSS weldments. Furthermore, nitrogen low cost gas compared to argon gas.

In this paper, the post weld heat treatment was performed at 1050 °C followed by water quenching to avoid formation of harmful phases (sigma, secondary austenite, chi, nitride, and carbides) which deteriorate microstructure, mechanical properties, and corrosion resistance.

## Experimental work

**Figure 1:**
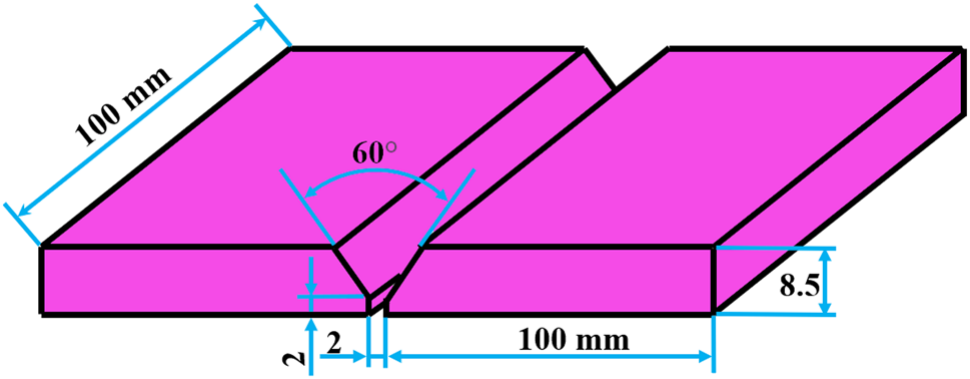
Schematic diagram of the V-groove geometry.

In the present work, DSS plates were welded using different welding processes at the same joint with a groove angle of 60°. Figure 1 shows the groove geometry used.

**Figure 2:**
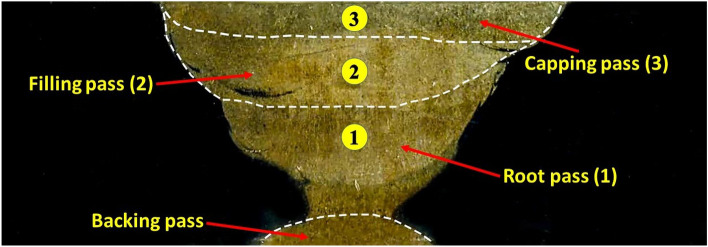
Macro section of a welded joint showing the sequences and the number of different passes.

Welding was carried out on industrial S32205 DSS hot rolled plates with 8.5 mm thickness and adopted dimensions of 100 × 100 mm (length × width). The welding process was carried out in root with shielded metal arc welding (SMAW) with Filler metal E2209-16 whereas the filling and capping were welded using gas tungsten arc welding (GTAW) with Filler metal ER2209 seen in Fig. 2. Table 1 shows the chemical composition of the DSS plate and different filler metals used in welding.

**Table 1:** The chemical composition of tested steel and filler metal.

Test material		C%	Si%	Mn%	P%	S%	Cr%	Ni%	Mo%	N%
Standard value of 2205 board max		0.03	1	2	0.03	0.02	22–23	4.5–6.5	3–3.5	0.14–0.2
Filler metal for GTAW	ER2209	0.02	0.4	1.7	–	–	22.5	8.8	3.2	0.15
Filler metal for SMAW	E2209-16	0.03	0.5	1.2	–	–	22.9	9	3.2	0.16

These filler metals were chosen because it has a similar chemical composition to that of the base metal (2205 DSS) seen in Table 1. In addition, the selected filler metals have a higher proportion of nickel compared to the base metal. Furthermore, nickel leads to increasing the volume fraction of the austenite phase in the welding zone therefore excellent resistance to stress, corrosion, cracking, and pitting^16^.

Also, shielding metal arc welding (SMAW) was used to substitute the backing gas and ensure high-quality welding in the filling and capping. The process parameters for both shielding metal arc welding (SMAW) and gas tungsten arc welding (GTAW) are shown in Table 2. One of the resulted welded joints is shown in Fig. 2.

**Table 2:** Welding process parameters.

SMAW (first pass)		GTAW (second and third pass)	
Specification number	WP-4025	Specification number	WP-4026
Welding current	115 A	Welding current	180 A
Welding voltage	28 V	Welding voltage	16 V
Welding speed	127 mm/min	Welding speed	100 mm/min
Size of filler metal	3.2 mm	Size of filler metal	2.4 mm
–	–	Shielding gas	Pure Argon (mixture 100%)
No. of passes	1 Pass (initial pass)	No. of passes	2 Passes
Initial and interpass cleaning	Wire brushing, grinding	Initial and interpass cleaning	Wire brushing, grinding

After welding the cap and root were removed using a milling machine with cutting fluid, then the samples were cut using a wire cutting machine.

**Figure 3:**
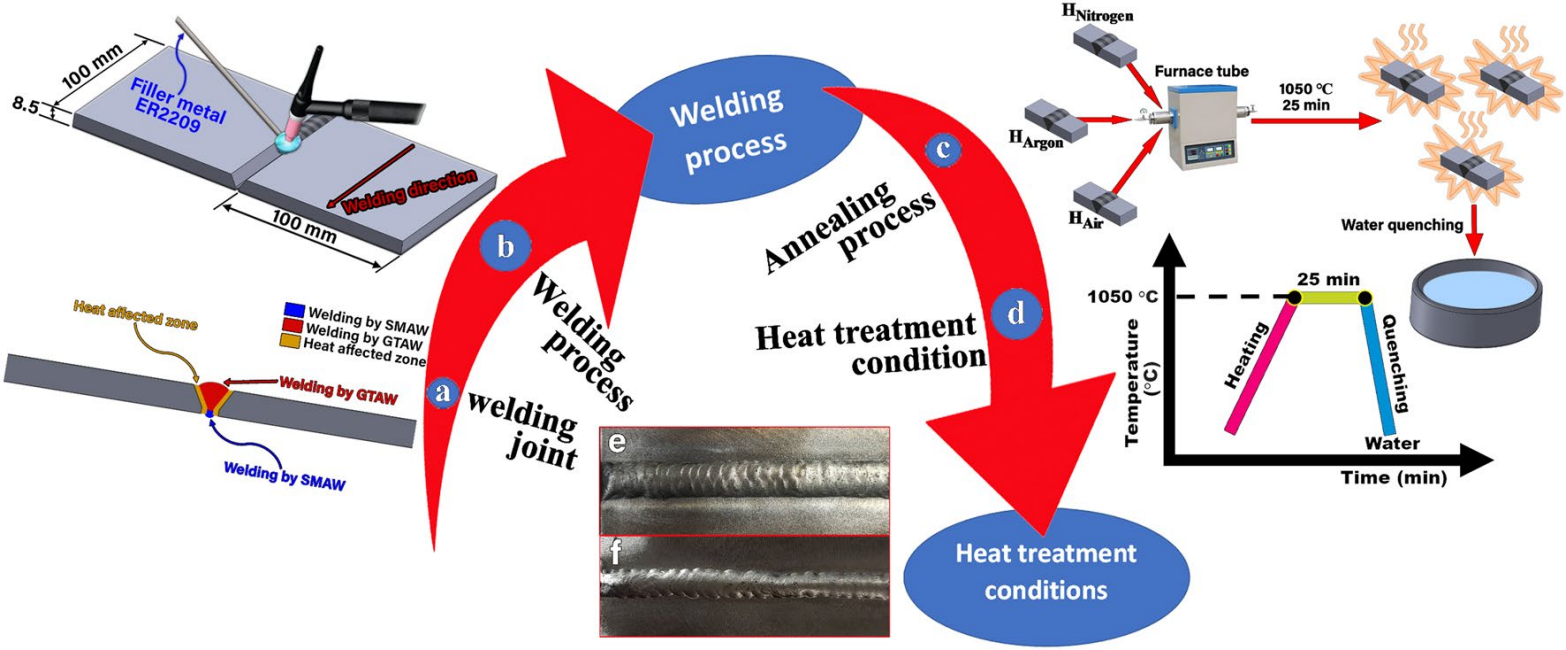
(a) Schematic welding joint, (b) schematic welding process, (c) schematic annealing process, (d) heat treatment condition, (e) cap of welding, and (f) root of welding.

To investigate the effect of heat treatment atmosphere after PWHT on the microstructure evolution, the pitting corrosion resistance, mechanical properties, and the secondary phase precipitation, the samples were annealed at 1050 $^\circ{\rm C}$ for 25 min, and then water quenched seen in Fig. 3. Post weld heat treatment (PWHT) was carried out in a tube furnace using different atmospheres (argon and nitrogen, and without protection gas). Table 3 shows the post weld heat treatment conditions used.

**Table 3:** Proposed post weld heat treatment conditions.

Test samples	Code	PWHT conditions		
		Temperature	Time	Gas protection
As welded	W	–	–	–
PWHT with argon as a protection gas	H_Argon_	1050	25 min	Argon
PWHT with nitrogen as a protection gas	H_Nitrogen_	1050	25 min	Nitrogen
PWHT without protection gas	H_Air_	1050	25 min	–

In addition, to observe the microstructure an optical microscope (OM) was used, and the specimens were polished with diamond paste to 0.25 µm and ground from 400, 600, 800, 1000, 1200, 1500 to 2000 grit SiC abrasive paper successively. Then electrolytic etching in potassium hydroxide (KOH) solution (20 g potassium hydroxide (KOH) and 100 mL deionized water applying 7 V for 10–15 s) was used.

Potassium hydroxide (20% KOH solution) for electrolytic etching was used because it has excellent ability to highly attack different phases (ferrite, austenite, and sigma) and highly distinguish between them by good contrast of gray, white and dark, respectively, based on optical metallography. This was used to calculate the volume fraction of austenite and ferrite by Image J software. MATLAB software was used to calculate volume fraction of ferrite, austenite and secondary phases clearly^28^. Oxalic acid also was used to show the secondary austenite or intermetallic.

Tensile tests were carried out to observe ultimate tensile strength (UTS), proof stress (PS), elongation, and tensile coefficient.

The Vickers hardness test was performed in the polished samples (W, H_Air_, H_Argon_, and H_Nitrogen_) been performed was tested force of 1 kg, and the test force duration time was 15 s. Hardness values were measured in three zones (BM, HAZ, WZ) and average values were taken.

In order to evaluate the effect of heat treatment atmosphere during PWHT on the pitting corrosion of weld zone. All measurements were carried out with IviumStat electrochemical analyzer by using three electrodes are reference electrode (RE), platinum foil used as a counter electrode (CE), and saturated calomel electrode (SCE). In addition, the specimens were polished with diamond paste to 0.25 µm and ground from 400, 600, 800, 1000, 1200, 1500 to 2000 grit SiC abrasive paper successively and an electrochemical corrosion solution was used from 3.5% NaCl.

## Results and discussion

### Microstructure of base metal

**Figure 4:**
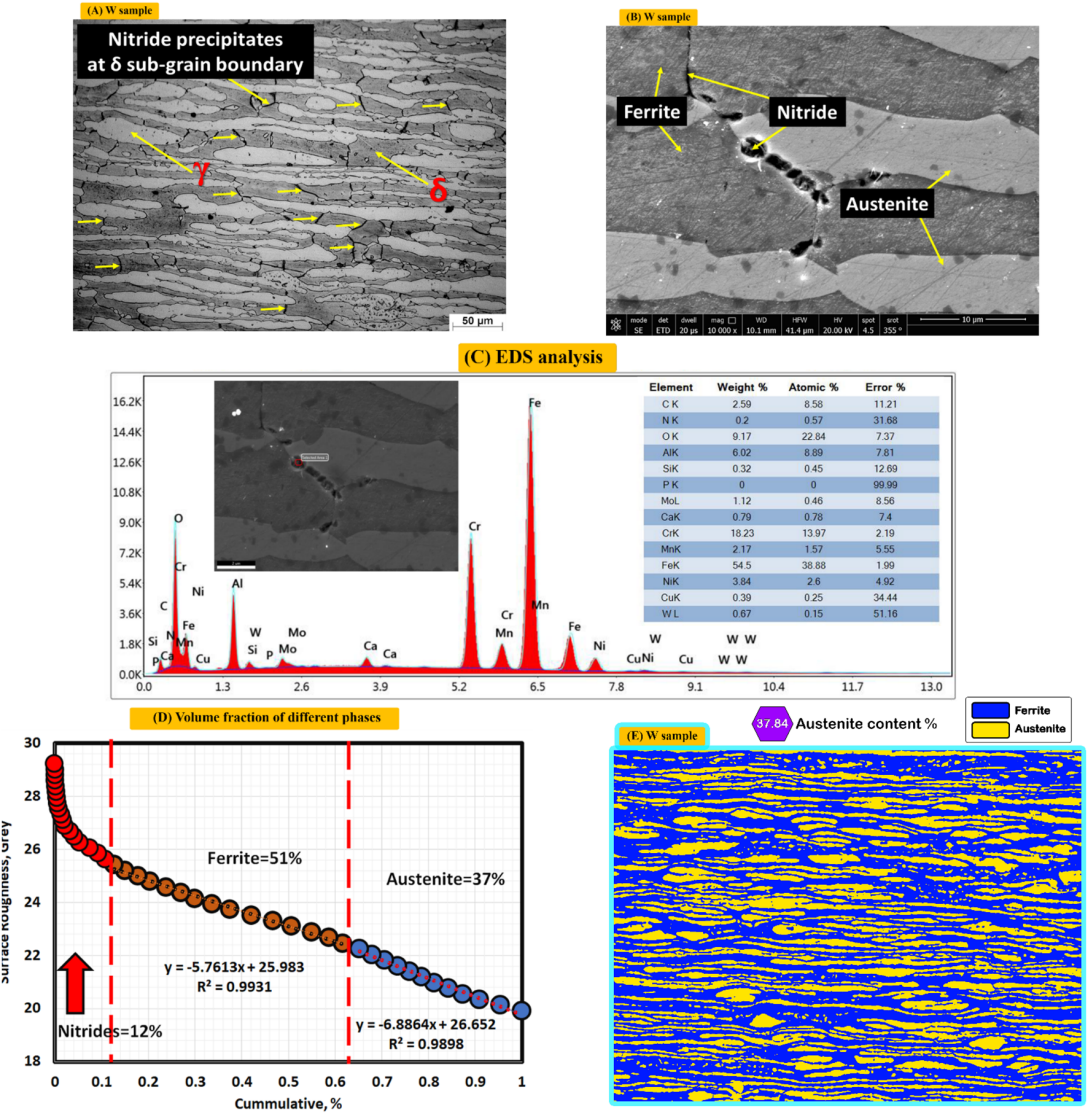
Optical, SEM micrograph, EDS analysis and different volume fraction of different phases for base metal, (**A**) optical microstructure, (**B**) SEM photo, (**C**) EDS analysis, (**D**) volume fraction by MATLAB, and (**E**) volume fraction by Image J.

Figure 4A shows optical microstructure of base metal. It consists of F (Grey), A (elongated or banded white structure) and nitride precipitates at δ sub-grain boundary (fine black points or lines between ferrite grains). Figure 4B shows the SEM photo where nitrides clearly appear as black points.

EDS reveals that high amount of nitrogen content and correspondingly, shows decreased Chromium content is existed in selected analyzed EDS point (chromium nitride), see Fig. 4C. Figure 4D shows volume fraction of different phases by MATLAB software on the other hand, Fig. 4E shows volume fraction of austenite phase by Image J software, where shows rapprochement of volume fraction austenite phase with MATLAB software.

### Microstructure of weld zone

**Figure 5:**
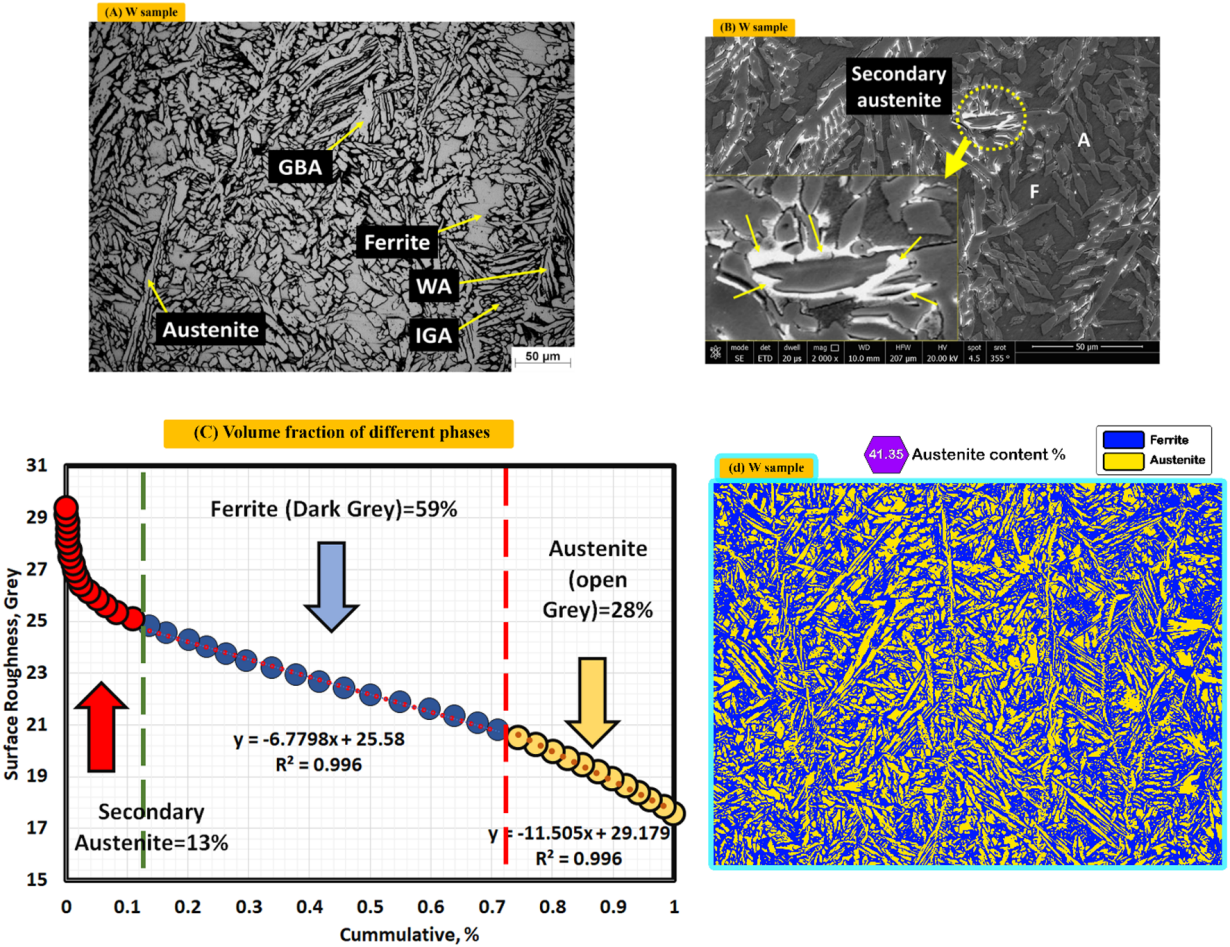
Optical, SEM micrographs and different volume fractions for weld zones of Sample (W) without PWHT process, (**A**) optical microstructure, (**B**) SEM photo, (**C**) volume fraction of different phases by MATLAB software, and (**D**) austenite content of one random image of weld zone by image J software.

**Figure 6:**
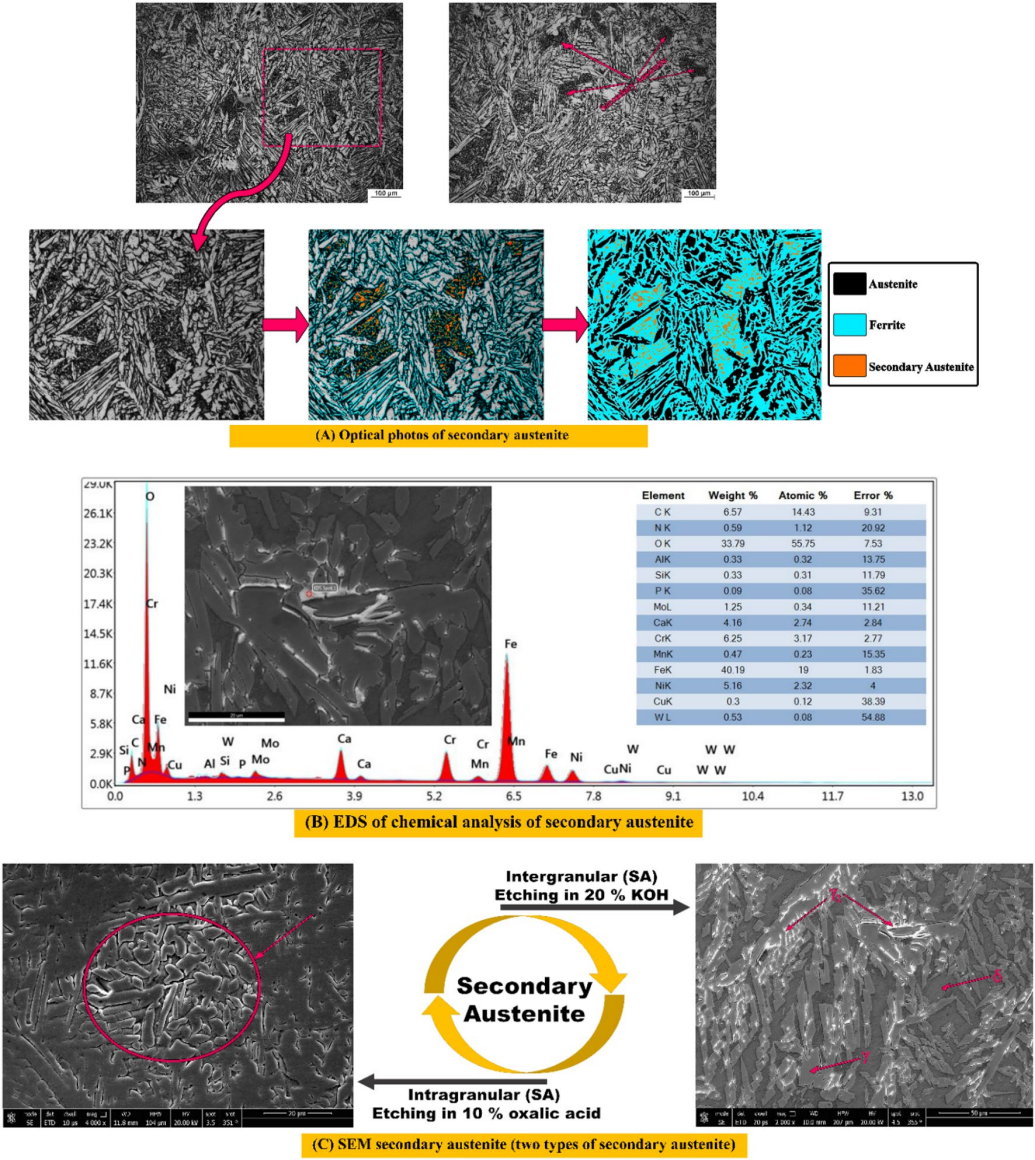
Optical, SEM micrographs of weld zones and EDS analysis point at secondary austenite of W sample (without PWHT process).

Nomenclatures for microstructure of weld zones of as welded is (W) and the three post-weld heat treated symbols during air, nitrogen and argon are H_air_, H_nitrogen_, and H_argon_, respectively. It is noticed in the as welded sample (W) exhibits different types of austenite. They are called grain boundary austenite (GBA), Widmanstattten austenite (WA), and Intergranular austenite (IGA). The GBA grows at the ferrite grain boundaries, then WA grows from GBA, also IGA nucleates at ferrite grain that contains high Ni concentration as shown in Fig. 5^12^. Also, the secondary austenite appeared in microstructure, the clear evidence of secondary austenite is EDS results which reveal that a high nickel, calcium, aluminum, and oxygen contents. Correspondingly, shows drastically decrease Chromium and molybdenum contents in the selected analyzed EDS point as seen in Fig. 6B^6,8,14,29^.

On the other hand, a high percentage of ferrite (59%) was existed in as-welded condition sample as seen in Fig. 5C,D.

Figure 5A shows optical microstructure of weld zone which contains GBA, F, A, WA and IGA. However, the optical photo does not show secondary austenite (SA). Therefore, it was necessary to use SEM photo to demonstrate secondary austenite (SA), see Fig. 5B. The secondary austenite was formed as seen in Fig. 5B due to using multi pass welding where multi pass technique allows to form excessive amount of secondary austenite^14^.

Figure 6A shows (SA) using optical and SEM microstructure. EDS reveals that high amount of Ni, Ca, Al, and O contents and Correspondingly, shows drastically decrease Cr and Mo contents are existed in selected analyzed EDS point (secondary austenite), see Fig. 6B. Furthermore, there are two types of (SA), first one is intergranular secondary austenite while second one is intragranular secondary austenite as seen in details in Fig. 6C. It is well known that secondary austenite suffers from low corrosion resistance^14^.

**Figure 7:**
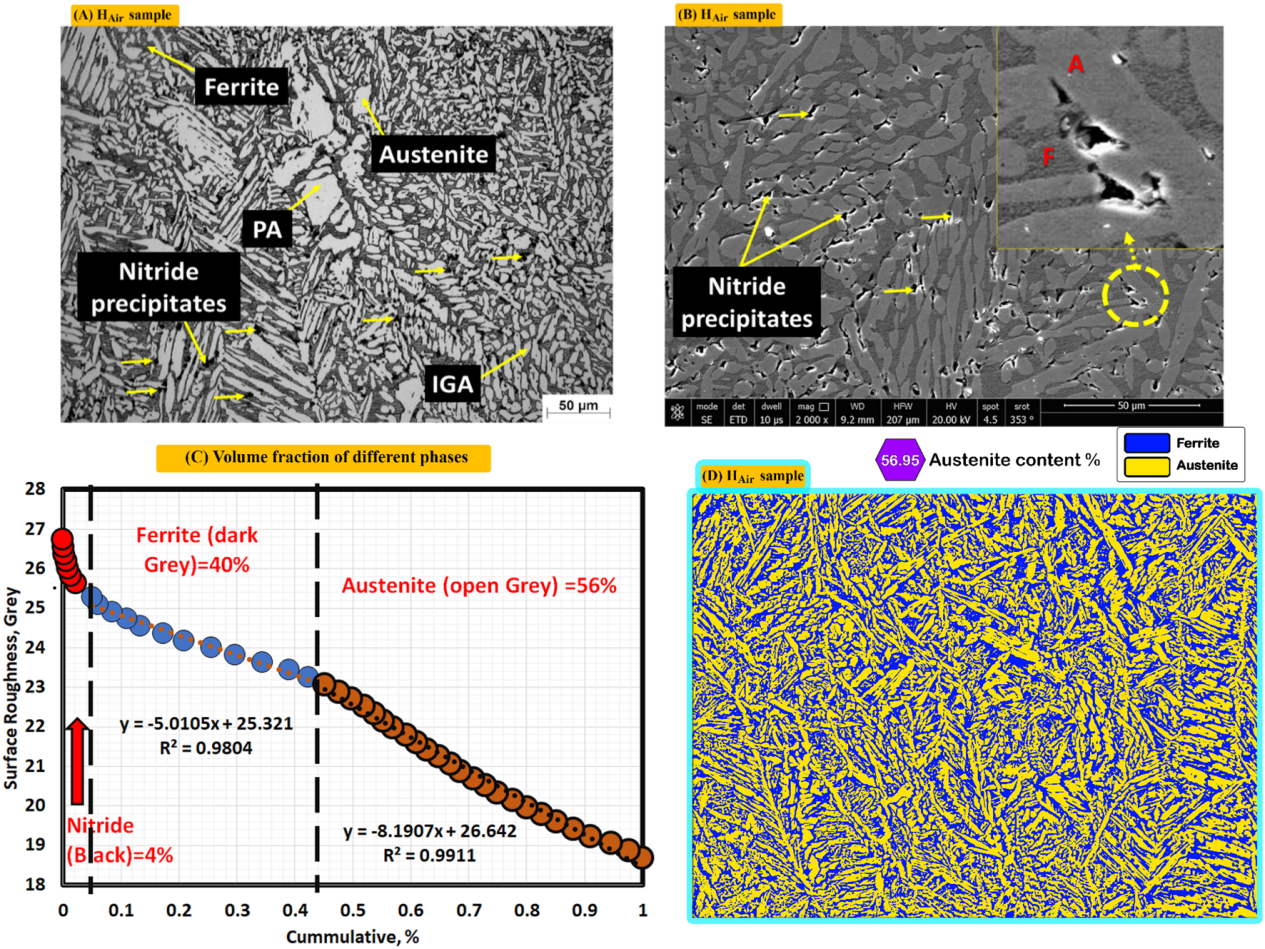
Optical, SEM micrographs and different volume fractions for weld zones of sample (H_Air_) not protected during PWHT process (**A**) optical microstructure, (**B**) SEM photo, (**C**) volume fraction by MATLAB software, and (**D**) austenite content of one random image of weld zone by image J software.

Figure 7A shows optical microstructure of non-protected sample (sample was subjected to PWHT without any protection gas). Optical microstructure consists of F (Grey), PA (white), IGA (fine structure) and nitride precipitates (black points). Optical microstructure also shows columnar austenite (dendritic structure). Figure 7B shows SEM photo where nitrides clearly appear as black points. Figure 7C demonstrates different volume fraction of F (40%), A (56%) and nitrides (4%) by MATLAB software. Figure 7D shows volume fraction of A (56.95) by Image J software.

**Figure 8:**
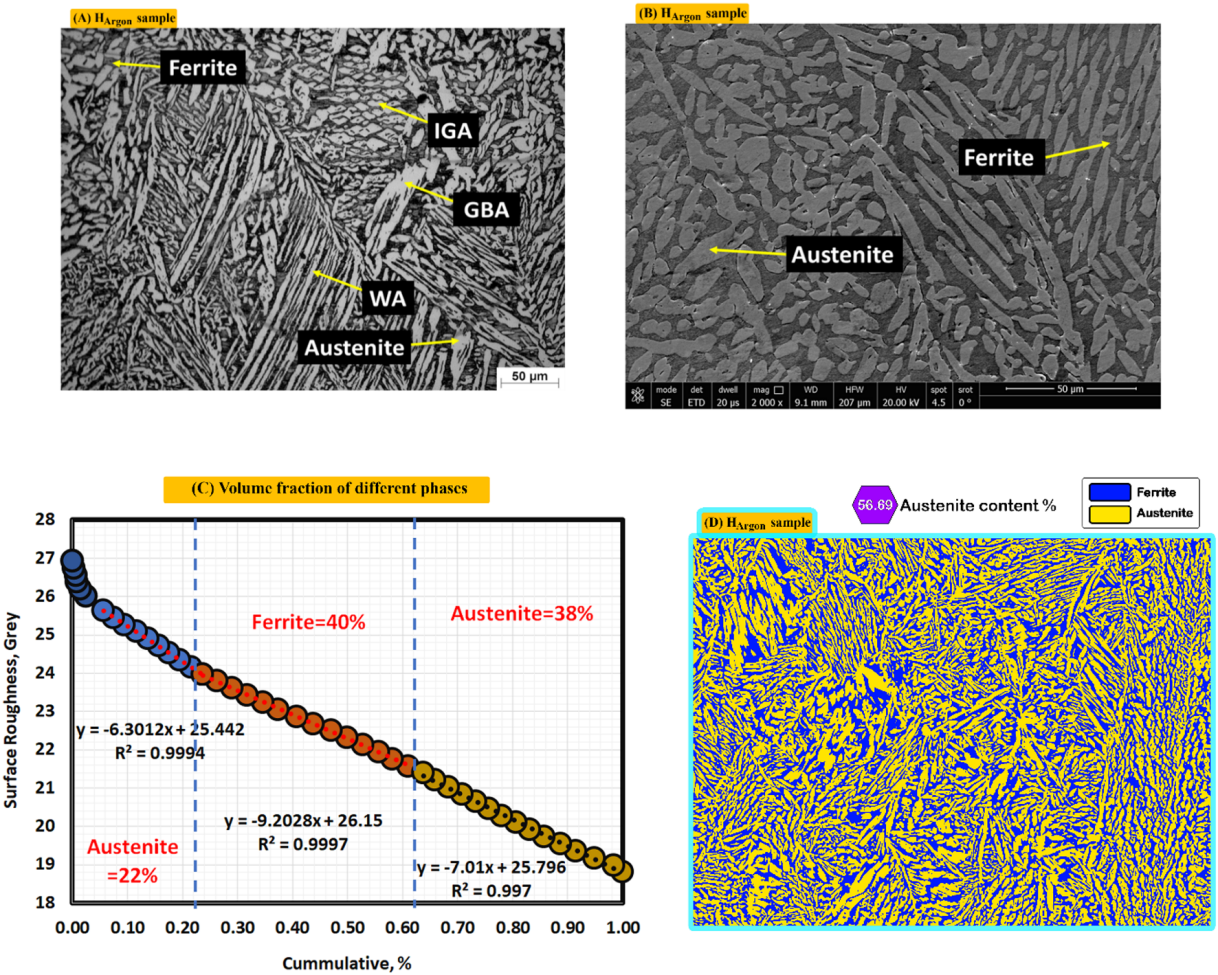
Optical and SEM micrographs of weld zones of sample (H_Argon_) protected with Argon gas during PWHT process (**A**) optical microstructure, (**B**) SEM photo, (**C**) volume fraction by MATLAB software, and (**D**) austenite content of one random image of weld zone by image J software.

Figure 8A shows optical microstructure of argon gas protected sample (H_argon_). Microstructure consists of F (Grey), A (columnar in white), IGA (fine structure). Figure 8B shows SEM photo where no nitrides existed, also, there is no any obvious intermetallic. Figure 8C shows different volume fraction of A (60) and F (40%) by MATLAB software. Figure 8D shows volume fraction of A (56.69) by Image J software.

**Figure 9:**
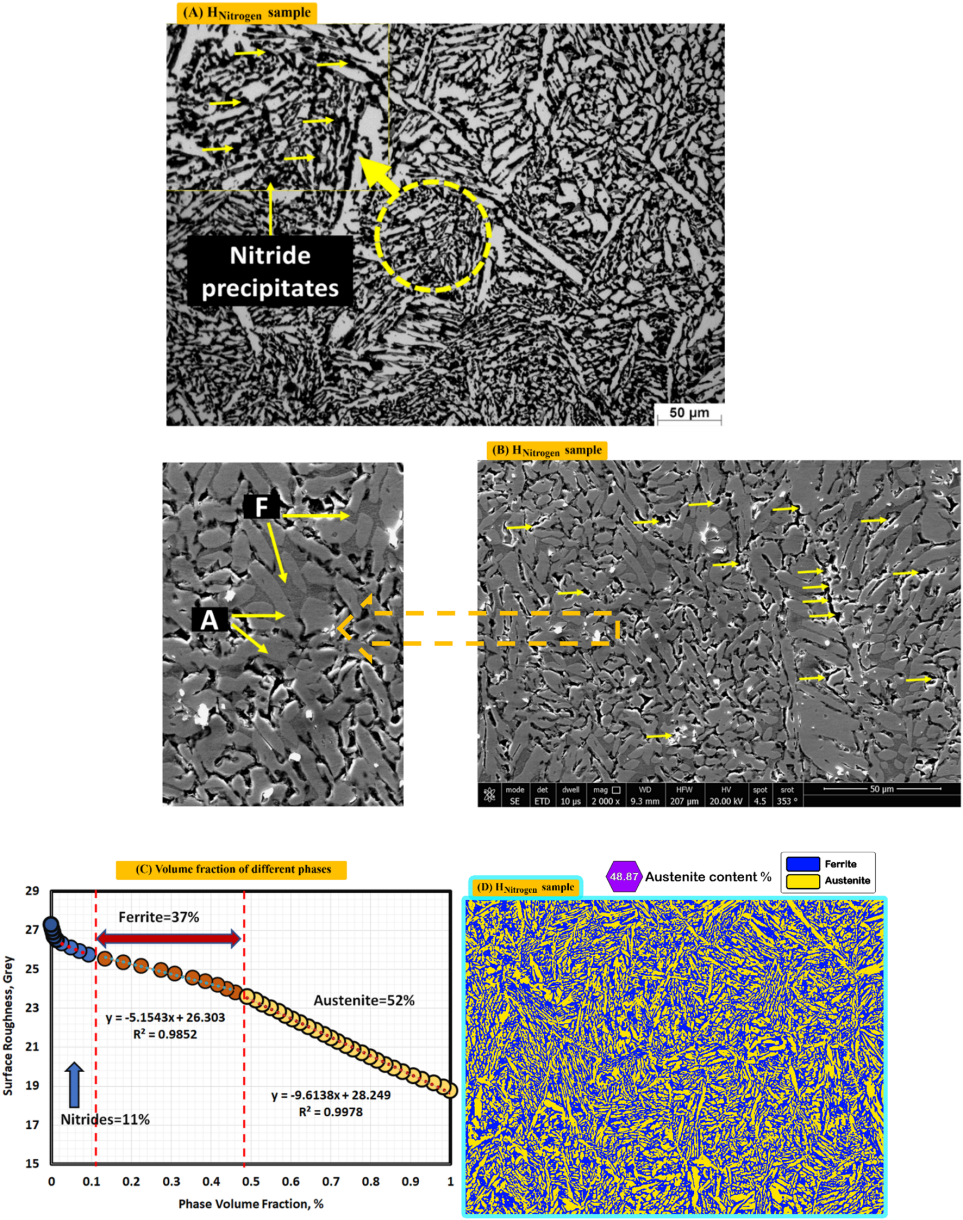
Optical, SEM micrographs and different volume fraction for weld zones of sample (H_Nitrogen_) protected with Nitrogen gas during PWHT process (**A**) optical microstructure, (**B**) SEM photo, (**C**) volume fraction by MATLAB software, and (**D**) austenite content of one random image of weld zone by image J software.

Figure 9A shows optical microstructure of nitrogen gas protected sample (H_Nitrogen_). It consists of few amounts of F (Grey), A (coarse and columnar in white), IGA (fine structure) and nitride precipitates (black points). Figure 9B shows the SEM photo where a lot of nitrides are existed in black while Ferrite is in Grey. Different volume fraction by MATLAB software of F, A and nitrides are 37, 52 and 11%, respectively as seen in Fig. 9C. As Fig. 9D shows volume fraction of A (48.87) by Image J software.

### Microstructure of heat affected zones (HAZ)

**Figure 10:**
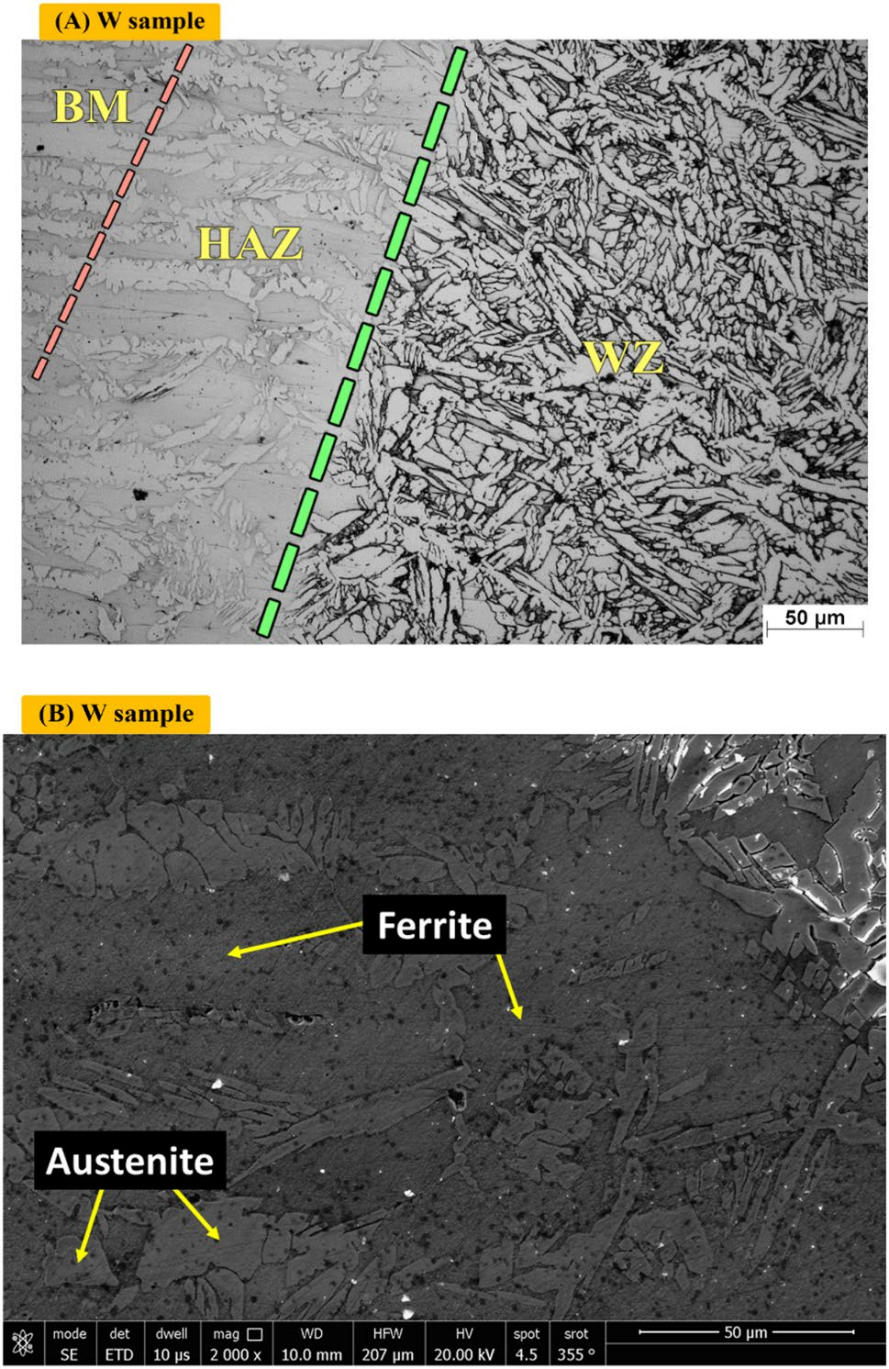
Optical and SEM micrographs of heat effected zone of sample (W) without PWHT process.

Figure 10A shows optical microstructure of heat effected zone (HAZ) of Sample (W) which contains F, A. Figure 10B shows SEM of heat effected zone (HAZ) of Sample (W) without PWHT process. It consists of Austenite and Ferrite.

Austenite (open color and more white), light dark as in left side is Ferrite.

**Figure 11:**
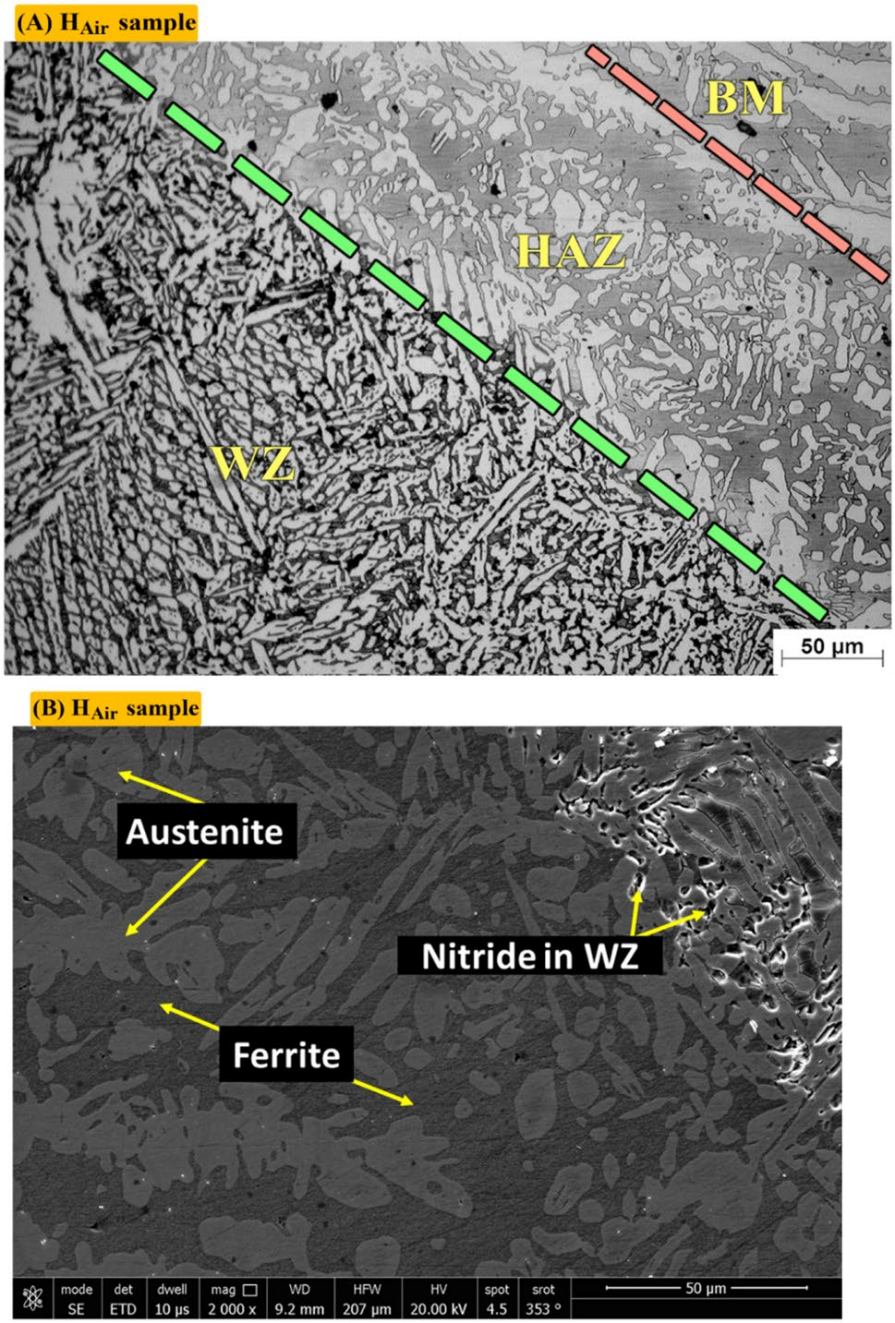
Optical and SEM micrographs of Heat effected zone of sample (H_Air_) not protected during PWHT process.

Figure 11A shows optical microstructure of Heat affected zone (HAZ) of Sample (H_Air_) of non-protected sample with PWHT process which contains F (light dark), A (white). Figure 11B shows SEM of heat effected zone (HAZ) of Sample (H_Air_). It consists of Ferrite and Austenite.

**Figure 12:**
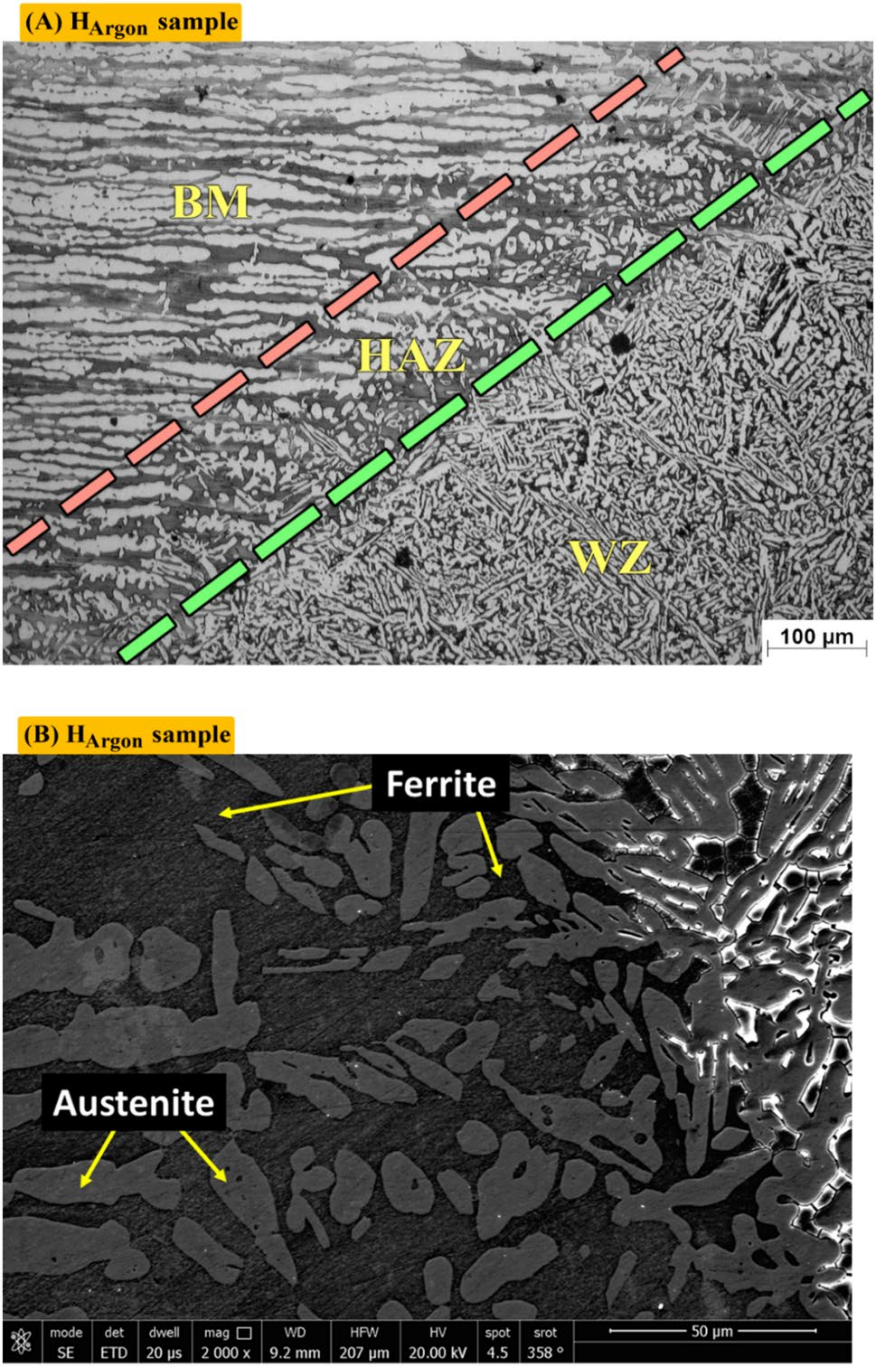
Optical and SEM micrographs of Heat effected zone of sample (H_Argon_) protected with Argon gas during PWHT process.

Figure 12A shows optical microstructure of Heat effected zone (HAZ) of sample (H_Argon_) protected with Argon gas during PWHT process which contains F (dark), A (white). Figure 12B shows SEM of heat effected zone (HAZ) of sample (H_Argon_). It consists of Ferrite (dark) and Austenite (Grey).

**Figure 13:**
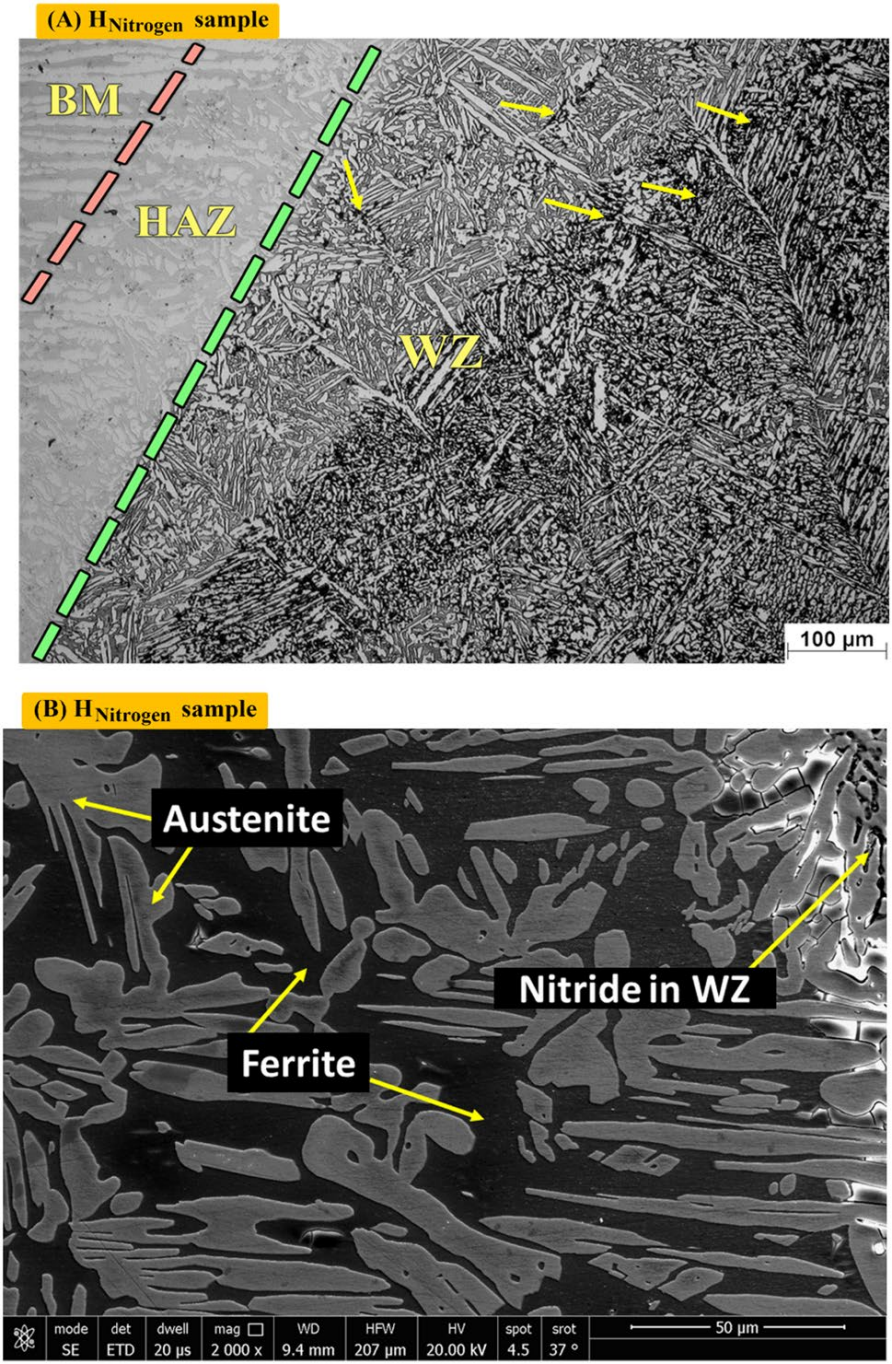
Optical and SEM micrographs of Heat effected zone of sample (H_Nitrogen_) protected with Nitrogen gas during PWHT process.

Figure 13A shows optical microstructure of Heat effected zone (HAZ) sample (H_Nitrogen_) protected with Nitrogen gas during PWHT process which contains F (grey), A (white). Figure 13B shows SEM of heat effected zone (HAZ) of Sample (H_Nitrogen_) in details. It consists of Ferrite (dark) and austenite (Grey).

**Figure 14:**
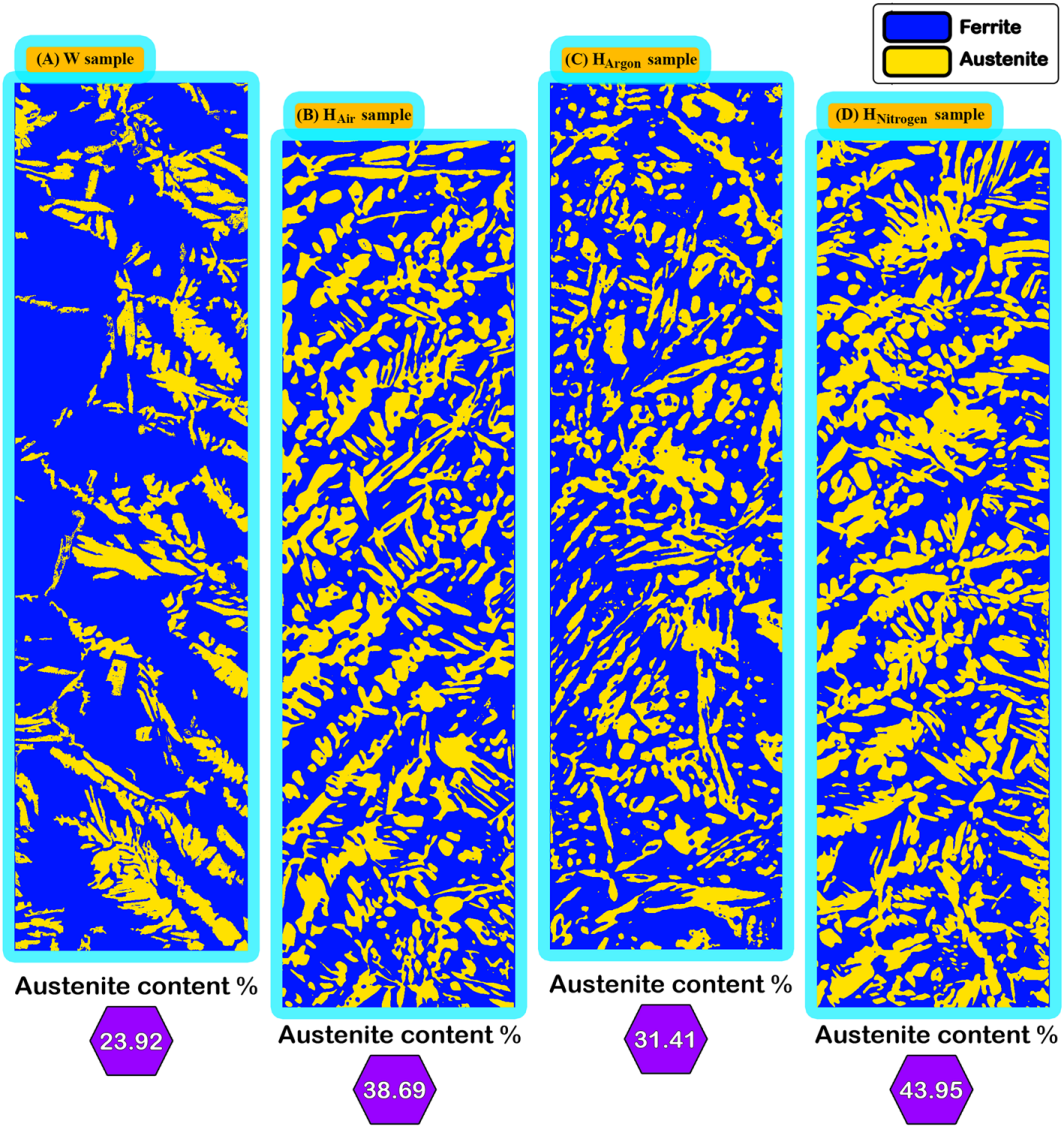
Volume fraction of austenite phase of one random image of heat affected zone of Samples (W, H_Air_, H_Argon_, and H_Nitrogen_).

Figure 14 shows the austenite content (austenite volume fraction) for one image of the heat affected zone of (W, H_Air_, H_Argon_, H_Nitrogen_) samples by image J software. A clear reduction of austenite fraction and consequently increases of ferrite content is observed in the (W) sample compared the heat treated samples. and in order of samples from where the highest of austenite volume fraction is (H_Nitrogen_, H_Air_, H_Argon_, and W) respectively.

### HAZ–WZ interface

**Figure 15:**
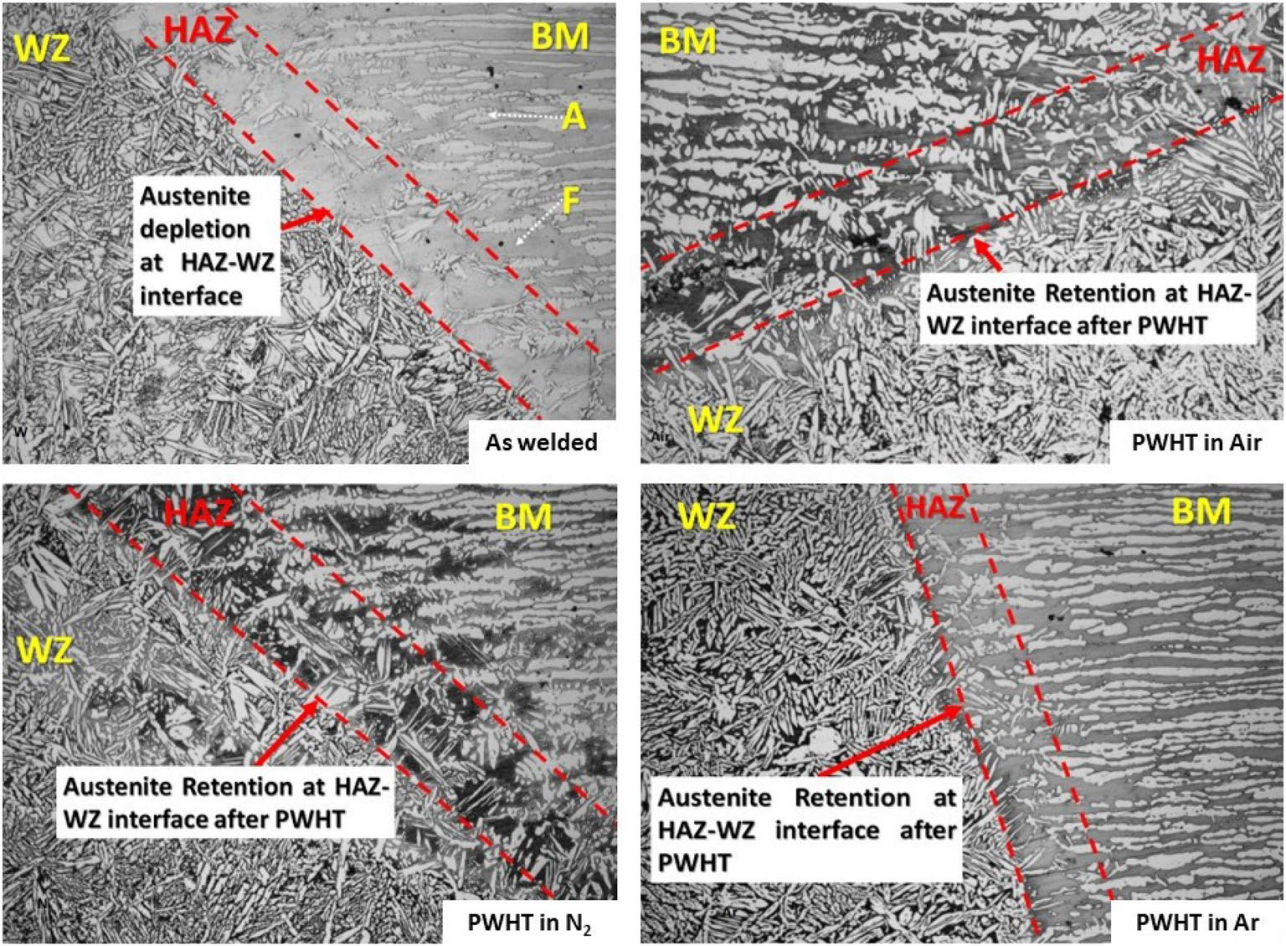
HAZ–WZ interface of (W, H_Air_, H_Argon_, H_Nitrogen_).

Figure 15 shows a depletion of austenite fraction in the heat affected zone at the interface adjacent to the weld pool whereas an increase of austenite fraction is observed at these interfaces after PWHT. The interruption of austenite/ferrite balance in the duplex stainless steels may deteriorate the properties, especially the corrosion resistance. Therefore, there may be an urgent need to restore this balance when it is disturbed due to the welding process, and PWHT will then have an important role to restore this balance. On the other hand, an increase in the ferrite grain size at the HAZ–WZ interface for the welded sample without PWHT which can be produced from the high heat input produced by welding process^30^. Moreover, one of the most important factors that may control the HAZ microstructure is reheating process due to multiple passes welding techniques^6,30,31^.

**Figure 16:**
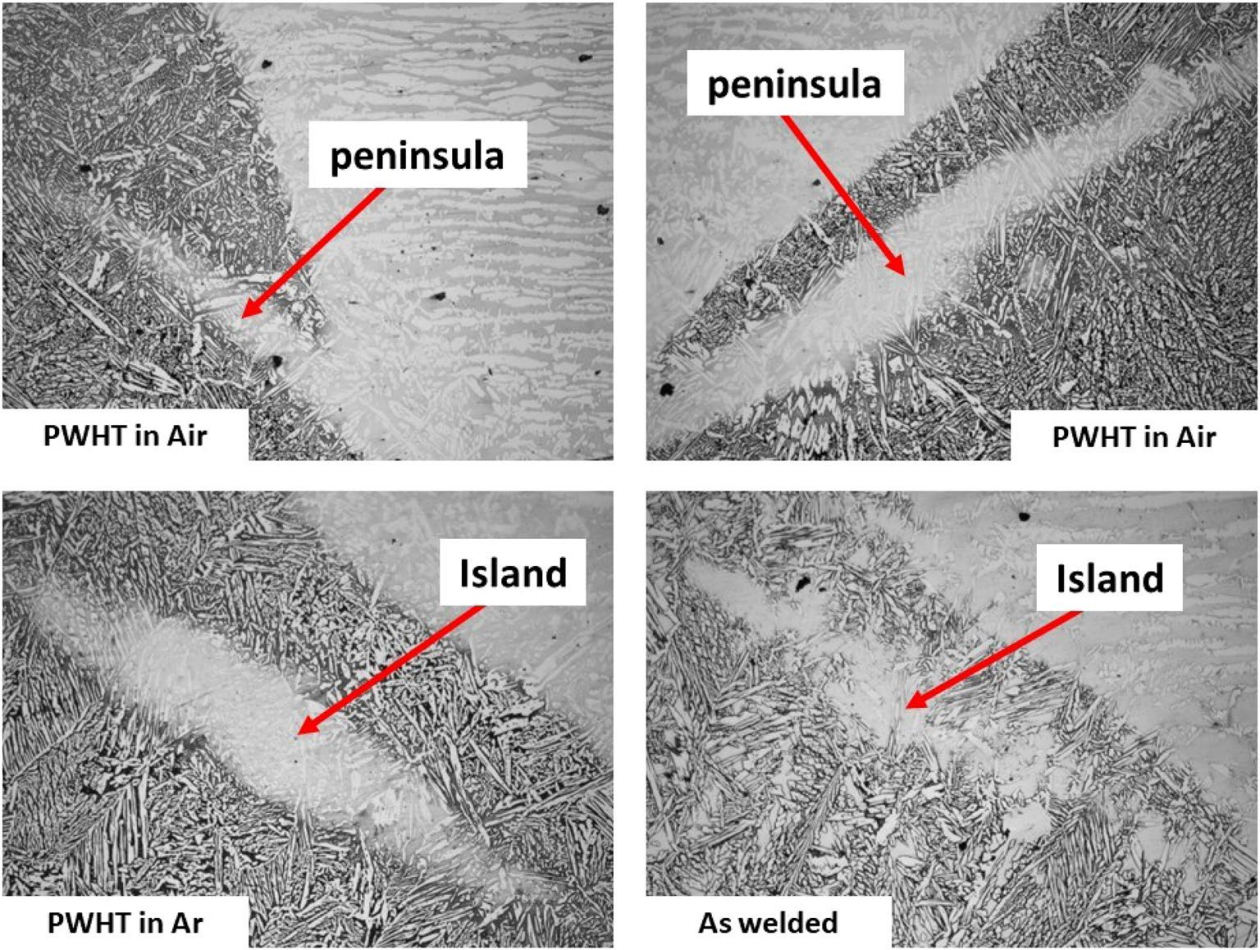
Fusion boundary (HAZ–WZ interface).

Because of the difference between filler metal and base metal in the melting point macrosegregation is formed near the fusion boundary. Macrosegregation takes different forms such as Transition zone (TZ), Unmixed zone (UZ), and Partially mixed zone (PMZ). Several studies indicate that nickel mixing during the welding process leads to the formation of a gradient microstructure near the fusion boundary (HAZ–WZ interface)^32,33^. In addition, there are different types to describe Unmixed zone (UZ), first one is island while the second one is peninsula in addition third one is filler deficient beach^32,33^. Figure 16 shows the formation of a gradient microstructure (island and peninsula) near the fusion boundary.

### Austenite volume fraction measurements

**Figure 17:**
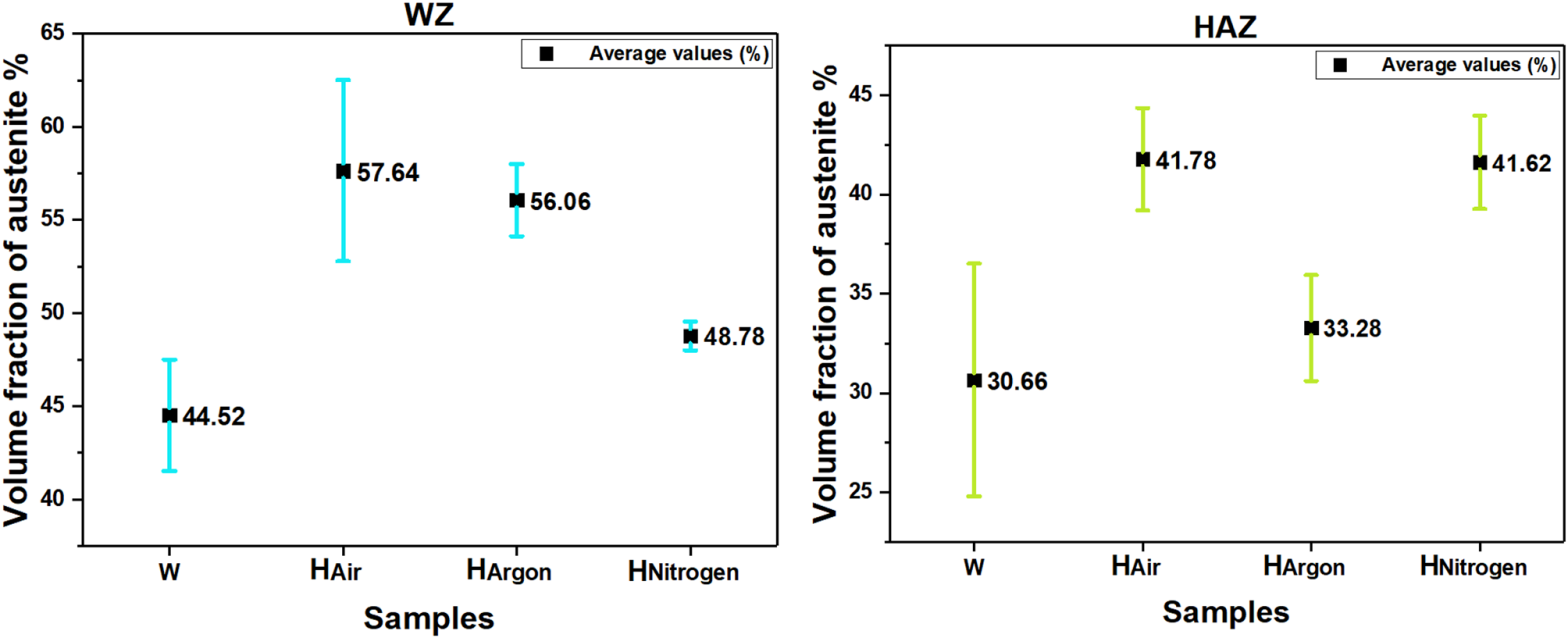
The volume fraction of austenite phase in heat affected zone and weld zone for the investigated specimens.

Figure 17 shows austenite volume fraction in the investigated samples. Because of the contrast in colors between austenite, ferrite, and intermetallic phases. Measurements were carried out by Image J software to calculate the volume fraction of the ferrite and austenite phases. Considering the proportion of the volume fraction of the ferrite phase includes the volume fraction of intermetallic phases, and because of the inhomogeneous distribution of alloying elements inside DSS. Five images of random locations in each zone (HAZ and WZ) were measured. A clear reduction of ferrite fraction and consequently increases of austenite content is observed in the heat treated samples compared to the as-welded (W) sample. Among with the heat treated samples at different atmospheres the sample treated under nitrogen atmosphere (H_Nitrogen_) reveals the lowest austenite content of weld zone. Furthermore the highest austenite content of weld zone is observed in the sample without protected atmosphere heat treatment (H_Air_ sample). Furthermore, that austenite volume fraction of Heat affected zone of (H_Air_ and H_Nitrogen_) samples was convergent.

### Nitride precipitation

The effect of post weld heat treatment on the properties of duplex stainless steel welded joints was studied by several researchers. In the present wok two types of controlled atmospheres are used individually that are argon and nitrogen compared with non-protected sample. Most of heat treatment processes are used argon as controlled atmosphere and the using nitrogen as controlled atmosphere for post weld heat treatment of duplex stainless steel is nearly not found in the publications. Using nitrogen (purity 95%) instead of argon as controlled atmosphere has an economic considerations because nitrogen gas is less expensive than argon gas^34^. The microstructural investigations for heat treated samples indicate that using argon as controlled atmosphere results in no second phase precipitation whereas second phase precipitation is observed in microstructure of sample under nitrogen atmosphere. Also, the unprotected sample (air is the furnace atmosphere) gives intermetallic precipitates somewhat similar microstructure of protected one with nitrogen. Nitrogen gas is classified as an inert gas and probable interaction with duplex stainless steel during heat treatment is out of the question. On the other hand, Brunzel et al.^35^ studied the effect of nitrogen gas on iron alloys during annealing. They noticed an increasing of nitrogen content (in the form of nitrides) in steel after annealing process compared with the starting material before annealing. Also, they concluded the intensity of the effect of nitrogen gas to form nitrides depends mainly on temperature–time parameters of the process, and the composition of the atmosphere, as well as the contents of alloying elements in the steel. In the present work, nitride precipitates are detected in the ferrite regions for samples heated without controlled atmosphere and that heated using nitrogen controlled atmospheres whereas sample treated in the argon controlled atmosphere reveals no precipitation. This is agreeing with the results of Brunzel et al.^35^ where nitride precipitation is observed. Interaction of molecular nitrogen and the iron alloys obeys the reaction:

$${\text{N}}_{2} \rightleftarrows 2{\text{N}}_{{{\text{Fe}}}} .$$

That has an equilibrium constant:

$$Kp=\frac{{a}^{2}N}{P{N}_{2}},$$

where a $N$ is the thermodynamic activity of nitrogen in solid solution of iron, and P_N2_ is the partial pressure of nitrogen in the atmosphere.

The amount of nitrogen dissolved in the iron depends on the temperature, nitrogen partial pressure, and the form and contents of alloying elements in the alloy in equilibrium with the gaseous atmosphere^36^. Moreover, the solubility of nitrogen in austenite is greater than that in ferrite and with increasing of temperature the nitrogen solubility decreases in austenite and increases in ferrite^37^.

### Tensile test results

**Figure 18:**
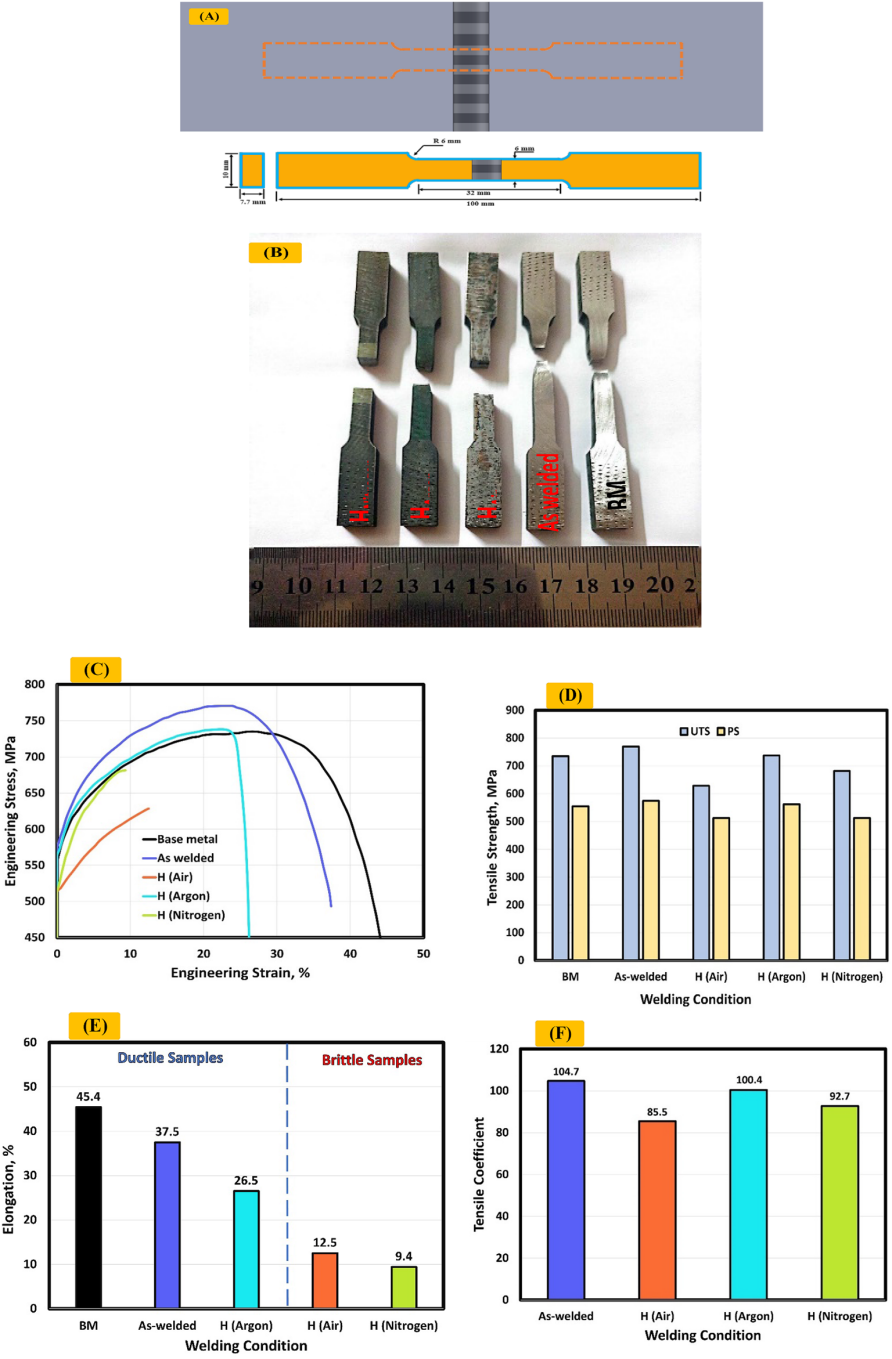
(**A**) Dimensions of tensile samples, (**B**) fractured tensile specimens, (**C**) engineering stress–strain curves of (W, H_Air_, H_Argon_, H_Nitrogen_) samples, (**D**) value of ultimate tensile strength and proof, (**E**) percentage elongation of samples, (**F**) tensile coefficient.

Tensile testing was performed to observe ultimate tensile strength (UTS), proof stress (PS), elongation, and tensile coefficient. Figure 18A shows the dimensions of tensile samples.

Figure 18C shows engineering stress–strain curves of (W, H_Air_, H_Argon_, H_Nitrogen_) samples. Results indicate the ultimate tensile strength of base metal was 734.9 MPa while an observed increase in UTS reached 4.7% and 0.4% for as welded and H_Argon_ samples respectively. The ultimate tensile strength of as welded and H_Argon_ samples was 769.3 MPa and 737.8 MPa respectively Fig. 18D. In addition, A clear decrease is observed in the UTS reaching 14.5% and 7.3% for H_Air_ and H_Nitrogen_ samples respectively. The ultimate tensile strength of H_Air_ and H_Nitrogen_ samples was 628.4 MPa and 681.4 MPa seen in Fig. 18D.

Furthermore, proof stress (PS) were 554.3, 574.3, 512, 561.7, and 512.3 MPa for base metal, as welded, H_Air_, H_Argon_, and H_Nitrogen_ samples respectively shown in Fig. 18D.

Figure 18B shows fractured tensile specimens. Observed location of the failure of as welded sample away of weld zone. In addition, the failure occurred to in weld zone of PWHT samples.

Figure 18F shows tensile coefficient of base metal, as welded, H_Air_, H_Argon_, and H_Nitrogen_ samples. Tensile coefficient was 104.7, 85.5, 100.4, and 92.7% for as welded, H_Air_, H_Argon_, and H_Nitrogen_ samples respectively.

Figure 18E shows the percentage elongation of samples. In terms of elongation a significant reduction is noticed for H_Air_ and H_Nitrogen_ samples. This reduction of ductility may be attributed to the formation of nitrides in H_Air_ and H_Nitrogen_ sample. In addition, harmful phases make duplex stainless steel was prone to Embrittlement^4^ consequently low corrosion resistance and deteriorated mechanical properties^19,20,23,26,27^. Confirms it engineering stress–strain curves seen in Fig. 18C and the percentage elongation seen in Fig. 18E deteriorated mechanical properties for H_Air_ and H_Nitrogen_ samples due to formation of nitride. Correspondingly, an observed drop in H_Argon_ sample compared to as welded sample. Bhanu^32^ indicated that the PWHT can negatively affect the ductility. Therefore, samples can be classified after tensile test to ductile and brittle seen in Fig. 18E.

### Hardness measurements values

**Figure 19:**
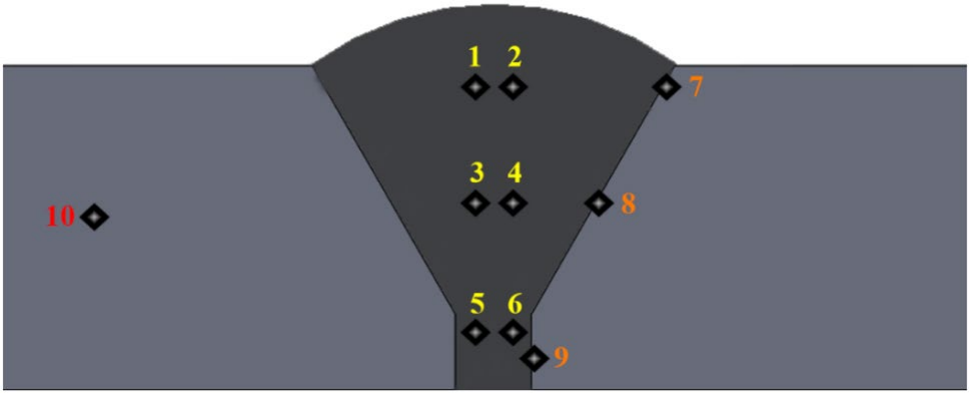
The zones where the hardness was measured in each sample.

**Figure 20:**
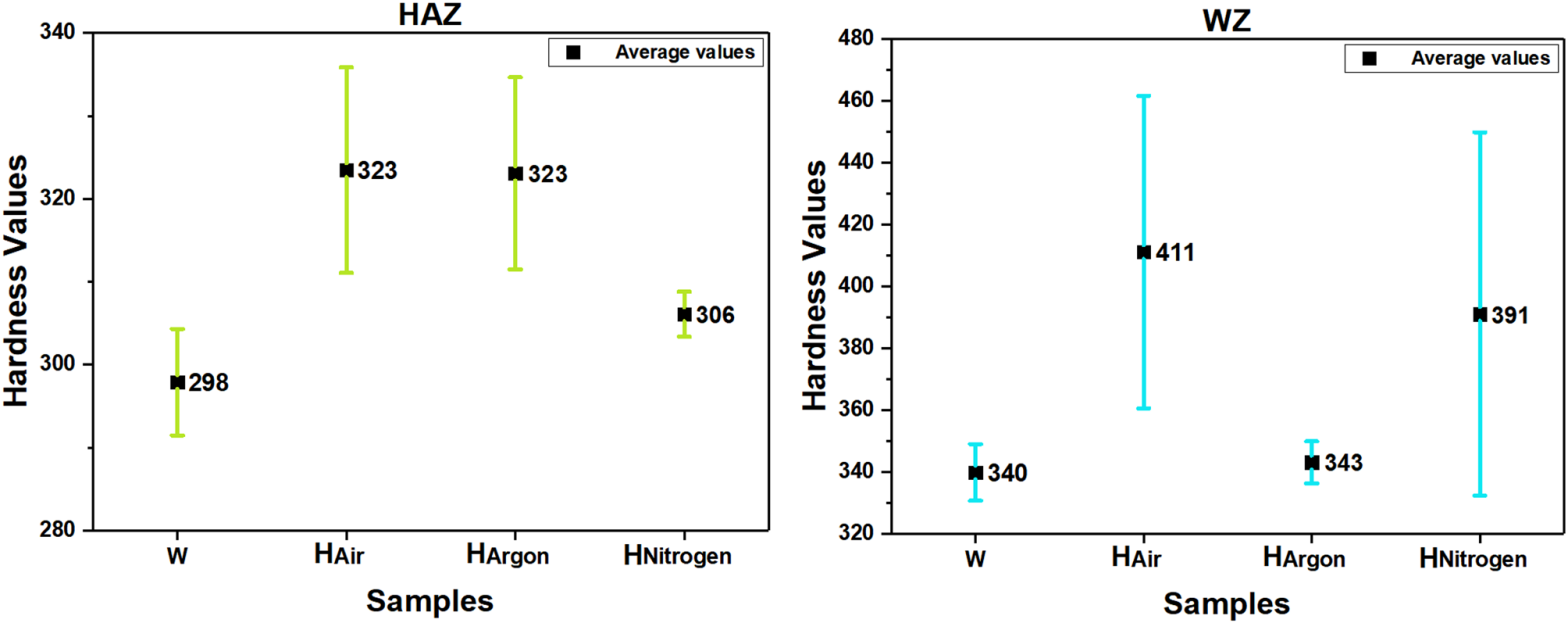
Average values of hardness of weld zone and heat affected zone.

Figure 19 schematic showing the locations of the measured hardness values. The average values of hardness for weld zone for samples W, H_Air_, H_Argon_, and H_Nitrogen_ were 340, 411, 343, and 391 respectively see Fig. 20. While average of hardness of heat affected zone was (298, 323, 323, and 306) for (W, H_Air_, H_Argon_, and H_Nitrogen_) samples respectively as shown in Fig. 20. Furthermore, hardness values of base metal were (286, 325, 317, and 304) of (W, H_Air_, H_Argon_, and H_Nitrogen_) samples respectively. Table 4 shows hardness values of samples in detail.

**Table 4:** Hardness values of samples in different 10 points in each sample.

Samples	WZ						HAZ			BM
	Cap		Middle		Root		Cap	Middle	Root	–
	1	2	3	4	5	6	7	8	9	10
W	343.9	338.6	325.3	334.1	350.4	346.8	291	299	303.7	286.2
H_Air_	445.7	456.3	432.2	438.9	340.9	352.8	314	318.9	337.5	325.3
H_Argon_	331.9	343.7	343.3	339.7	350.4	349.5	336.4	317.3	315.5	316.9
H_Nitrogen_	448.7	447.7	409.3	401.8	326.4	312.8	306.8	303.1	308.4	303.8
Test force				1 kg	–	Test force duration time				15 s

The apparent increase of hardness values for weld zone in the PWHA samples using air and nitrogen atmospheres may be resulted from the precipitation of nitrides. In addition, inhomogeneous distribution for alloying elements inside DSS^19^ and change in chemical composition at both ferrite and austenite phases (shown in Table 5) may also leads to variations of hardness values. Furthermore, effect of cooling rate and reheating during welding leads to different hardness values in different regions of weld zone.

**Table 5:** Chemical composition of austenite and ferrite phase for the test samples and PREN of WZ after four times EDS measurement.

Sample	Zone	Phase	Chemical composition (mass%)					PREN
			Mo%	Cr%	Mn%	Ni%	N%	
W	WZ	Austenite	2.55	25.26	1.42	8.88	0.31	38.635
		Ferrite	3.24	26.38	1.44	7.39	0.14	39.312
H_Air_	WZ	Austenite	2.29	24.68	1.47	8.33	0.25	36.237
		Ferrite	2.63	25.92	1.34	7.12	0.21	37.959
H_Argon_	WZ	Austenite	2.35	23.33	1.51	8.73	0.24	34.925
		Ferrite	3.09	25.72	1.47	6.47	0.18	38.797
H_Nitrogen_	WZ	Austenite	2.22	23.61	1.49	8.98	0.20	34.136
		Ferrite	2.39	25.62	1.48	6.73	0.14	35.747

### Electrochemical test

**Figure 21:**
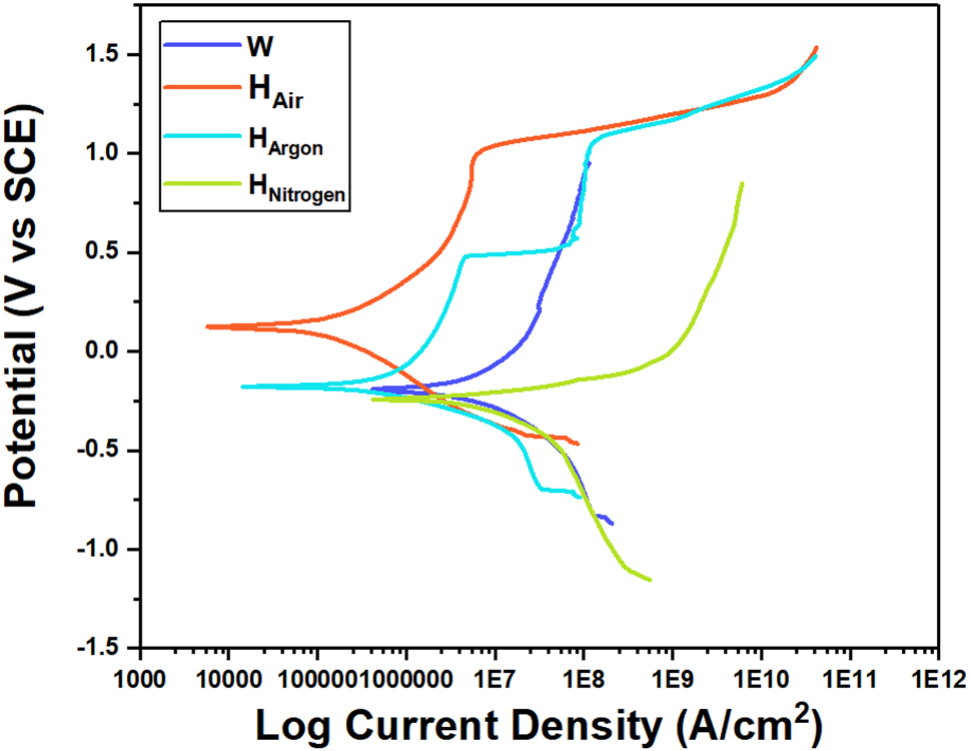
Polarization curves of weld zone of (W, H_Air_, H_Argon_, H_Nitrogen_) samples.

Figure 21 shows polarization curves of Tafel corrosion test of (W, H_Air_, H_Argon_, H_Nitrogen_) samples, polarization curves indicate exciting things. Although there is no any obvious intermetallic in H_Argon_ sample and precipitates (Nitride) presence in weld zone of H_Air_ sample. The highest corrosion resistance was (H_Air_, H_Argon_, H_Nitrogen_, W) respectively seen in Fig. 21, attributed it to volume fraction of austenite and ferrite where corrosion is being in ferrite firstly due to the sodium chloride (NaCl) solution used^38^. As presence high content of nickel and nitrogen in austenite lead to high corrosion resistance in austenite compared ferrite seen in Table 5. In addition, that, more ferrite volume fraction lead to faster pit diffusion rate in the specimens^27^. So it was necessary to investigation volume fraction of ferrite and austenite. Zhang et al.^28^ concluded that PWHT for duplex stainless steels weldments increases the austenite volume fraction (i.e. decreasing balanced ferrite volume fraction) leading to the improvement of corrosion resistance of the welded joints that is agree with the results of this article. But presence Nitrides significantly in Nitrogen sample and secondary austenite in W sample was main reason of increase corrosion rate in this samples^14,39^. As corrosion current density is the main factor to determine corrosion rate. So increase corrosion current density lead to fast corrosion rate. Table 6 shows corrosion current density value, where was Arrange the samples according to the slow corrosion rate as follows (H_Air_, H_Argon_, H_Nitrogen_, W) respectively.

**Table 6:** Electrochemical parameters of weld zone after potentiodynamic polarization measurements.

Sample	E_corr_	βa	βc	I_corr_	I_corr_	E_pit_	Rp
	(V)	(V)	(V)	(A)	(A/cm^2^)	(V)	Ω
W	− 0.1963	0.439	0.296	2.12E−06	6.12E−06	–	3.63E+04
H_Air_	0.1239	0.216	0.233	3.45E−08	9.938E−08	0.96	1.41E+06
H_Argon_	− 0.1716	0.183	0.116	1.03E−07	2.966E−07	1.01	3.00E+05
H_Nitrogen_	− 0.2417	0.063	0.137	1.09E−06	3.15E−06	–	1.72E+04

## Conclusions

The following are important findings from this study on the effect of heat treatment atmosphere on the microstructure of duplex stainless steel welded joints:

- PWHT enhance the grain refining and increasing of austenite fraction in the weld zone and HAZ.
- Using of nitrogen as protection gas during heat treatment leads to the formation of nitride precipitates the same result is also obtained when the specimens heat treated without protected gas whereas the using of argon gas during heat treatment gives no nitride precipitation.
- Among with heat treated specimens using different furnace atmospheres the specimen that heated with using nitrogen during PWHT has the highest ferrite volume fraction (lowest austenite volume fraction) compared with specimens that using argon and air during heat treatment.
- A significant drop of UTS and ductility are observed after PWHT especially when using air and nitrogen as heat treatment atmospheres.
- Higher Vickers hardness values are observed for H_Air_ and H_Nitrogen_ welded joints, and this may be due to nitride precipitates.
- Reduction of corrosion resistance is noticed for post weld heat treatment using nitrogen (H_Nitrogen_ sample) and as welded (W sample) owing to nitride precipitation and secondary austenite respectively.
- Finally, it is not recommended to perform PWHT for duplex stainless steel weldments if the mechanical properties are of great importance, while if the chemical properties and corrosion resistance are required to be improved, it is preferable to perform PWHT because it improves the corrosion resistance of duplex welds.

## Data Availability

In relation to the availability of raw data I want to inform you that this work is a part of a long research study, and the raw data will not be public at the moment but are available from corresponding author upon the request.
